# Adaptation of OXPHOS biogenesis to cellular requirements

**DOI:** 10.1002/pro.70763

**Published:** 2026-08-14

**Authors:** Sven Dennerlein, Peter Rehling

**Affiliations:** ^1^ Department of Cellular Biochemistry University Medical Center Göttingen Göttingen Germany; ^2^ German Center for Child and Adolescent Health (DZKJ) Göttingen Germany; ^3^ Cluster of Excellence “Multiscale Bioimaging: From Molecular Machines to Networks of Excitable Cells” (MBExC), University of Göttingen Göttingen Germany; ^4^ Max Planck Institute for Multidisciplinary Science Göttingen Germany; ^5^ Fraunhofer Institute for Translational Medicine and Pharmacology, Translational Neuroinflammation and Automated Microscopy Göttingen Germany

**Keywords:** Cytochrome *c* Oxidase, mitochondria, OXPHOS

## Abstract

Metabolic cues regulate the formation of the mitochondrial OXPHOS machinery. These regulatory processes are tightly linked to mitochondrial translation, proteolytic degradation of unassembled subunits, and the formation of supercomplexes, creating checkpoints at which nutrient availability, oxygen tension, and signaling pathways remodel OXPHOS content and activity. In particular, the cytochrome *c* oxidase (COX) assembly pathway is regulated at multiple steps of its biogenesis in response to cellular demands. COX consists of mitochondrially encoded catalytic core subunits and nuclear‐encoded accessory subunits whose coordinated expression, cofactor insertion, and incorporation into the COX enzyme result in optimized electron transport capacity. Consequently, COX assembly depends on numerous dedicated factors and protein isoforms, many of which are expressed in a tissue‐specific manner. Through these metabolically regulated processes, cells tune oxidative phosphorylation efficiency, limit reactive oxygen species production, and support context‐specific metabolic programs in development, adaptation, and disease.

## 1 INTRODUCTION

Mitochondria are essential cellular organelles that serve as central metabolic hubs involved in orchestrating diverse cellular processes such as fatty acid oxidation, amino acid metabolism, calcium buffering, and the generation of reactive oxygen species (ROS) (Wiedemann & Pfanner, 2017). In addition to these functions, mitochondrial ATP production is the central energy‐generating process in eukaryotic cells, driven by the oxidative phosphorylation (OXPHOS) system. The OXPHOS system is located in the inner mitochondrial membrane (IMM) and couples the transfer of electrons from the reduced cofactors NADH and FADH_2_ to molecular oxygen via a series of protein complexes known as the electron transport chain (ETC). The ETC is composed of four multisubunit complexes that facilitate electron flow while protons are pumped from the mitochondrial matrix into the intermembrane space (IMS), generating an electrochemical proton gradient, also called the proton‐motive force. The energy stored in this proton gradient is harnessed by ATP synthase (complex V), which allows protons to flow back into the matrix through its membrane‐embedded channel, driving the rotary mechanism of ATP synthesis from ADP and inorganic phosphate. Besides ATP production, the proton gradient also contributes to maintaining the mitochondrial membrane potential and drives metabolite transport across the inner membrane.

The mitochondrial OXPHOS complexes I, III, IV, and V are composed of subunits of dual genetic origin. Each mitochondrion contains own circular DNA (mtDNA), which encodes 13 key components of the human OXPHOS. However, the majority of OXPHOS subunits are encoded by nuclear genes, synthesized on cytosolic ribosomes, and subsequently imported into mitochondria by dedicated import machineries (Busch et al., 2023; Dimogkioka & Rapaport, 2025; Heller et al., 2026; Jackson & Becker, 2025; Jain et al., 2025; Nussberger et al., 2024; Özdemir & Dennerlein, 2024; Wiedemann & Pfanner, 2017). The terminal enzyme of the ETC is the cytochrome *c* oxidase (complex IV or COX). In human mitochondria, COX consists of 14 subunits, three of which are encoded by mtDNA, whereas 11 are nuclear‐encoded and must be imported into the organelle. While knowledge of tissue specific isoforms of the involved assembly factors is just emerging, seven core COX subunits exist in more than one isoform. These isoforms have been linked to the adaptation of complex IV functionality to metabolic demands or tissue‐specific conditions (Cabrera‐Alarcón et al., 2025; Popovic et al., 2025).

Focusing on the human COX enzyme, we will provide an outline of how COX assembly is integrated into the cellular context, followed by a snapshot of our current understanding of the COX assembly pathway and highlight the adaptation of the COX composition to metabolic demands and tissue‐specific contexts, focusing on the different COX core‐subunit isoforms.

## 2 COORDINATION OF OXPHOS ASSEMBLY IN THE CELLULAR CONTEXT

For decades, mitochondria were often investigated as isolated entities within cells, although many mutations, linked to mitochondrial disorders, have been described (Fernandez‐Vizarra & Zeviani, 2021). In recent years, it has become increasingly clear that mitochondria can adapt to pathological conditions and that mitochondrial function directly influences and reshapes cellular metabolism. To achieve this, mitochondria are integrated into different cellular stress pathways. These pathways reaching from transcriptional regulations over different protein distribution or protein relocation events. To intiate cellular stress responses involving mitochondrial dysfinction, signals can occur within mitochondria or at thire vicinity, for example during import defects. For example, in yeast leads an accumulation of mitochondrial precursor proteins in the cytosol to the intiation of the mPOS (mitochondrial Precursor Over‐accumulation Stress) pathway (Wang & Chen, 2015). Alterativly, provokes the limitation of mitochondrial import sites, for example, using clogger constracts, which compete with endogenous import substrates, a global transcriptional response (Boos et al., 2019). Accordingly, leads the over expression of mitochondrial yeast proteins to the indcution of the mitochondrial compromised protein import response (mitoCPR) (Weidberg & Amon, 2018) and the unfolded protein response activated by mistargeting of proteins (UPR^am^) (Wrobel et al., 2015). These in yeast described pathways are complemented by in human cells described pathways such as the mitochondrial integrated stress response (mitoISR) (Bao et al., 2016; Costa‐Mattioli & Walter, 2020; Croon et al., 2022; Quirós et al., 2017) or the mitochondrial unfolded protein response (UPR^mt^) (Nargund et al., 2012; Sutandy et al., 2023; Wrobel et al., 2015) Activation of these mitochondrial stress‐related pathways involves distinct cellular stress sensors and initiates global changes in cellular metabolism, the proteome, and the transcriptome to counteract stress conditions (Popovic et al., 2025; Sutandy et al., 2023; Wrobel et al., 2015). Furthermore, a pronounced tissue‐specific regulation upon mitochondrial dysfunction has been reported, indicating that different organs respond differentially to similar mitochondrial insults (Boshnakovska et al., 2025; Khan et al., 2017; Kutschka et al., 2023; Quirós et al., 2017).

In the context of mitoISR and the COX enzyme, a recent study by Popovic et al. (2025) provided insight into tissue‐specific adaptations. The COX specific assembly factor COX10, which is higly conserved between human and yeast, acting early in the COX assembly process, is considered as involved in the heme A synthesis. Specifically, COX10 functions as farnesyl trasnferase, generating first heme O, which gets further converted by COX15 to heme A and subsequential integration into COX (Bareth et al., 2013; Barros et al., 2001, 2002; Barros & Tzagoloff, 2002). Using heart‐ and skeletal muscle‐specific COX10‐deficient mouse models, the authors observed clear differences between these tissues upon COX10 loss. In the heart, glutathione metabolism and lipid biogenesis were markedly altered (Ahola et al., 2022; Popovic et al., 2025), whereas COX10 knockout in skeletal muscle did not induce mitoISR under the experimental conditions, despite a drastic reduction in cytochrome *c* oxidase levels (Popovic et al., 2025). Earlier studies reported that COX10 loss in skeletal muscle leads to a later onset of atrophy (after 3 months) (Diaz et al., 2005) and that sustained OXPHOS deficiency can eventually trigger mitoISR in skeletal muscle as well (Forsström et al., 2019). At present, the mechanisms underlying the distinct metabolic phenotypes in different tissues after COX10 depletion remain incompletely understood. One possible explanation is inter‐organ crosstalk between heart and skeletal muscle, whereby the heart supplies metabolites to muscle tissue, reducing the immediate need for mitoISR activation, or that the high regenerative capacity of skeletal muscle compared to heart tissue delays stress signaling (Ahola et al., 2022; Forsström et al., 2019).

However, an important aspect of tissue‐specific OXPHOS behavior could be the expression of protein isoforms. The contribution of isoforms to mitochondrial biogenesis in the tissue‐specific behavior, particularly for cytochrome *c* oxidase, is not well understood, although their expression levels are associated with various human disorders.

## 3 ASSEMBLY OF THE CYTOCHROME *C* OXIDASE: A SNAP SHOT

Cytochrome *c* oxidase is the terminal enzyme of the ETC and catalyzes the reduction of molecular oxygen to water. Structural analyses revealed that the mature enzyme comprises 14 subunits (Ishigami et al., 2023; Nguyen et al., 2026; Zong et al., 2018), whereas more than 30 accessory proteins are required for its assembly (Dennerlein et al., 2023; Fernández‐Vizarra & Ugalde, 2022; Timón‐Gómez et al., 2018). Three structural subunits (COX1, COX2, and COX3) are encoded by mtDNA. For proper enzyme functionality, COX1 contains heme a and heme a_3_, and COX1 (Cu‐B) and COX2 (Cu‐A) harbor copper centers, respectively (Ishigami et al., 2023; Nývltová et al., 2022; Zong et al., 2018). Therefore, the assistance of dedicated COX assembly factors is essential.

**FIGURE 1:**
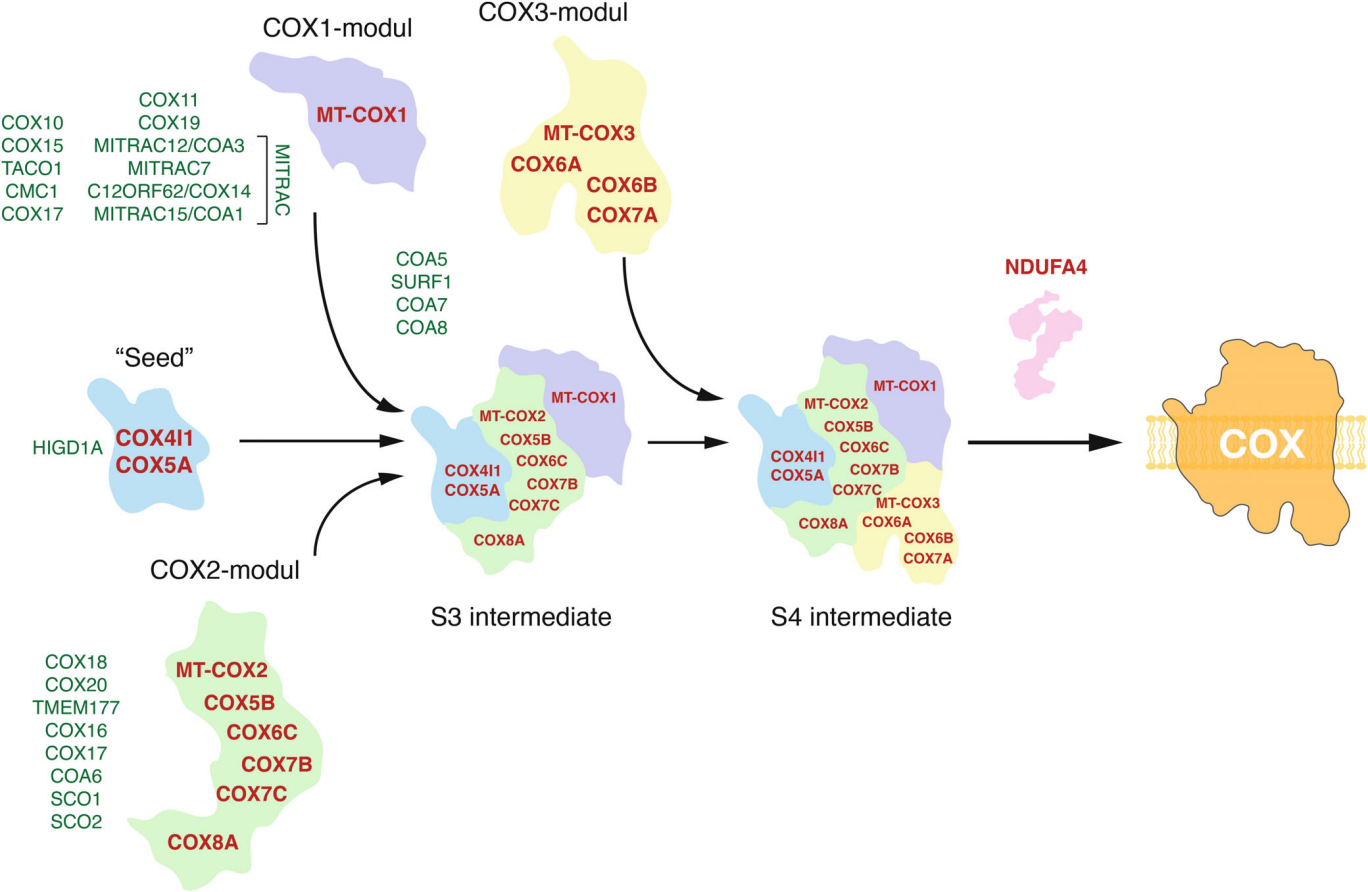
Modular assembly pathway of the monomeric cytochrome *c* oxidase. The current COX assembly model favors the modular formation of the COX enzyme. First, the “Seed”, the COX2‐ and the COX‐module are forming the S3‐intermediate on which the COX3‐module gets added (S4‐intermediate). The COX enzyme gets finalized by adding the NDUFA4 protein. Core subunits are depicted in red, corresponding assembly factors are green.

**FIGURE 2:**
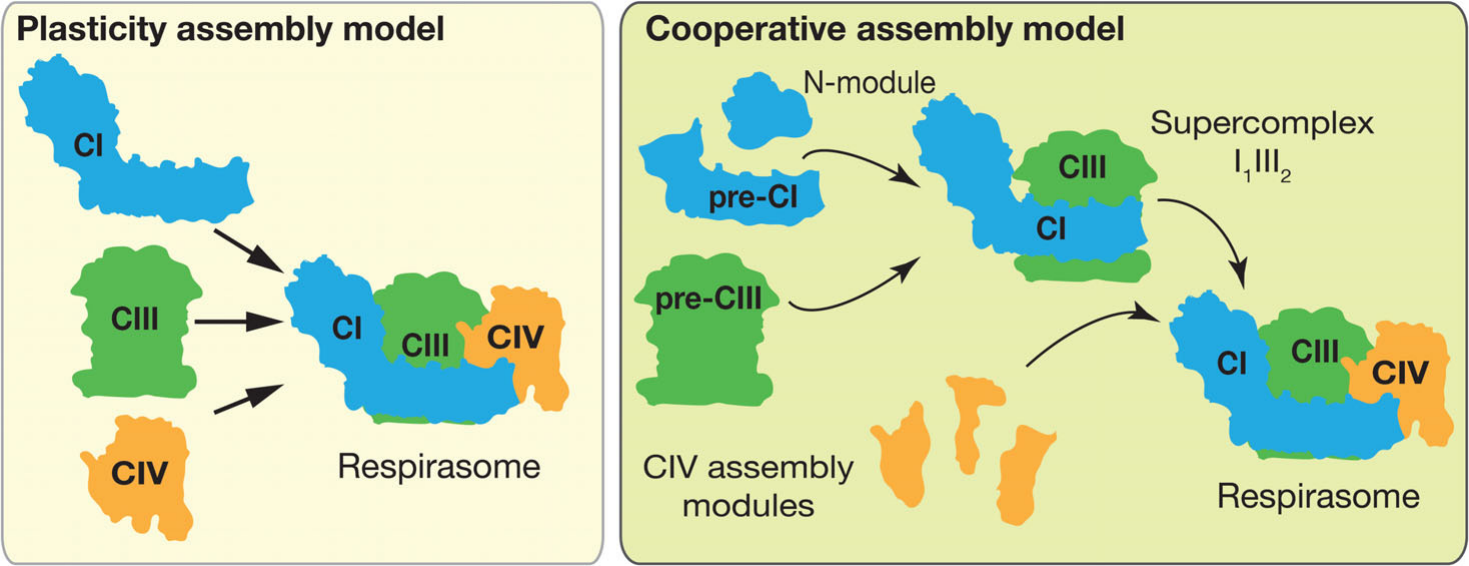
Schematic overview of the plasticity‐ and the cooperative assembly model. Within the plasticity model (left) complex I (blue), complex III (green) and complex IV (orange) are fully distinct assembled before they form the respirasome. The cooperative assembly model (right) is based on the assembly of structural subcomplexes of complex I (blue) and complex III (green) which join together in pre‐respirasome complexes before complex IV modules associate to form the respirasome.

It is now widely accepted that cytochrome *c* oxidase assembles in a stepwise, modular manner in which COX1, COX2, and COX3 each assemble first in distinct modules (Figure 1) (Brischigliaro & Zeviani, 2021; Fernández‐Vizarra & Ugalde, 2022; Lobo‐Jarne et al., 2020). These modules form independently and then associate to generate the mature monomeric enzyme. Currently it is believed that the COX1‐ and the COX2‐modul form together with the “seed” the S3‐intermediate on which the COX3‐modul gets associated (S4‐intermediate) (Figure 1). Finally, NDUFA4 assambles on it, finally forming the COX enzyme (Figure 1) (Brischigliaro & Zeviani, 2021; Fernández‐Vizarra & Ugalde, 2022; Lobo‐Jarne et al., 2020), which further incorporates into higher order respiratory chain supercomplexes (SCs). The timing and mechanism of SC formation are controversially discussed (Figure 2). One proposed “plasticity” model suggests that individual complexes are fully assembled before SCs are formed (Figure 2, left) (Acín‐Pérez et al., 2008), whereas the “cooperative” assembly model proposes that SC formation begins during the assembly of individual complexes (Figure 2, right) (Čunátová et al., 2026; Fang et al., 2021; Fernández‐Vizarra & Ugalde, 2022; Lobo‐Jarne et al., 2020; Mick et al., 2007; Protasoni et al., 2020; Vercellino & Sazanov, 2022). In addition, it has been suggested that pre‐existing complexes may serve as platforms for the assembly of newly synthesized respiratory complexes (Cogliati et al., 2016; Fang et al., 2021; Mick et al., 2007; Stroud et al., 2016). Blue‐Native gel analyses often reveals a large pool of the COX enzyme that is not incorporated into SCs, leading to the hypothesis that alternative assembly pathways exist (Lobo‐Jarne et al., 2020; Timón‐Gómez et al., 2018; Timón‐Gómez, Garlich, et al., 2020). Notably, incorporation of different COX isoforms appears to significantly influence SC formation, as observed for COX7A2/COX7A2L (Barrientos & Ugalde, 2013; Cogliati et al., 2016; Ikeda et al., 2013; Lapuente‐Brun et al., 2013; Mourier et al., 2014). A recent cryo electron microscopy study by Nguyen et al. (Nguyen et al., 2026) supports the idea that complexes I and III are fully assembled before cytochrome *c* oxidase becomes finalized by incorporation of NDUFA4, which itself also exists in different isoforms (Sinkler et al., 2017).

## 4 ADAPTATION OF HUMAN CYTOCHROME *C* OXIDASE COMPOSITION TO CELLULAR DEMANDS

Mitochondria are central for supplying biochemical energy in accordance with metabolic requirements and signaling pathways that determine cellular survival, differentiation, and adaptation to environmental changes. Several studies have implicated cytochrome *c* oxidase as a major regulator of OXPHOS activity, as ATP production is directly linked to COX function (Čunátová et al., 2026; Dennerlein et al., 2023; Fernández‐Vizarra & Ugalde, 2022; Nguyen et al., 2026; Sinkler et al., 2017; Stroud et al., 2016; Timón‐Gómez et al., 2018; Vogt et al., 2021). Subunit expression and assembly of COX are therefore thought to be tightly regulated (Čunátová et al., 2020, 2026; Nguyen et al., 2026). One important aspect is that COX biochemical properties depend on tissue type and metabolic state due to the expression and incorporation of distinct subunit isoforms (Pham et al., 2024; Popovic et al., 2025). Seven COX subunits (COX4, COX6A, COX7A, COX7B, COX8, and NDUFA4) exist in more than one variant, encoded by distinct genes (Čunátová et al., 2026; Pham et al., 2024; Sinkler et al., 2017).

### 4.1 COX4

**FIGURE 3:**
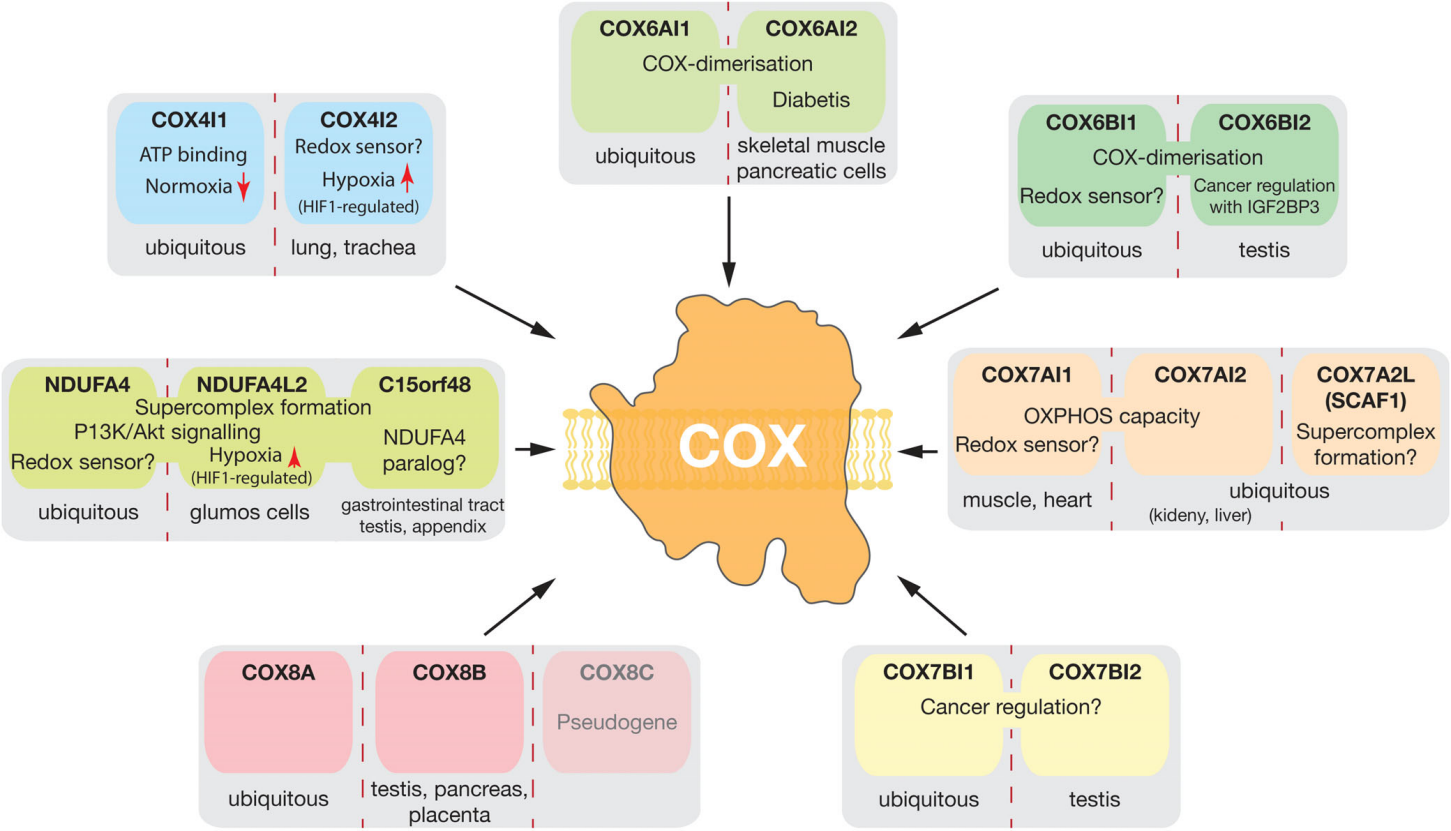
Overview of COX constituents with functions and tissue distribution. The COX enzyme contains seven subunits that are present in more than one isoform in humans. The individual proteins are illustrated and potential functions indicated. Overlapping functions are written below the individual isoforms in a common box. The corresponding tissues are indicated below each isoform (gray box).

Because cytochrome *c* oxidase transfers electrons to oxygen and produces water, it is not surprising that the expression and availability of different COX subunits, particularly COX4, have been linked to oxygen availability (Fukuda et al., 2007; Hüttemann et al., 2001; Pham et al., 2024). COX4 is the first nuclear encoded subunit that associates with COX1 during assembly (Nijtmans et al., 2022; Richter‐Dennerlein et al., 2016). Two human COX4 isoforms, COX4I1 and COX4I2 (Figure 3), were described in 2001 and are encoded by separate genes on chromosomes 16 and 20, respectively (Hüttemann et al., 2001). Comparative analyses suggest that COX4I2 arose approximately 320 million years ago, coinciding with an increase in atmospheric oxygen from about 13% in the Devonian age to approximately 35% during the Permian age, indicating evolutionary pressure to fine tune respiratory control (Dudley, 1998; Fukuda et al., 2007; Hüttemann et al., 2001; Takano et al., 2014).

COX4 directly binds ATP and thereby contributes to allosteric regulation of COX activity by sensing the cellular ADP/ATP ratio (Acin‐Perez et al., 2011; Arnold & Kadenbach, 1997; Hüttemann et al., 2001; Ludwig et al., 2001; Napiwotzki & Kadenbach, 1998). ATP binding to COX4 depends on the phosphorylation state of Ser^58^ in COX4I1, which has been proposed to be regulated by CO_2_, providing a potential link between COX activity and the Krebs cycle (Acin‐Perez et al., 2011). COX4I2 lacks Ser^58^ but contains three cysteine residues, which may form disulfide bonds and function as redox sensors (Hüttemann et al., 2001; Reguera et al., 2020). This notion is supported by increased COX4I2 expression and higher COX activity under hypoxia and reduced ROS production in COX4I2 containing enzymes (Fukuda et al., 2007).

The two COX4 isoforms show differential tissue expression (Figure 3) in relation to oxygen availability (Fukuda et al., 2007; Kocha et al., 2015; Sinkler et al., 2017; Timón‐Gómez, Garlich, et al., 2020). COX4I1 is ubiquitously expressed under normoxic conditions, whereas COX4I2 is highly expressed in hypoxic tissues, particularly lung and trachea, and to lower amounts in rat heart and brain (Douiev et al., 2021; Kocha et al., 2015; Sinkler et al., 2017; Timón‐Gómez, Garlich, et al., 2020; Wu et al., 2009). Mechanistically, COX4I2 gene regulation appears complex. Hypoxia‐induced expression has been attributed to a specific 24‐bp COX4I2 proximal promoter element (Hüttemann, Lee, et al., 2012) or due to HIF‐1 binding sites in the COX4I2 intron 1 (Fukuda et al., 2007). Additionally, hypoxia increases levels of the mitochondrial LON protease, which promotes degradation of COX4I1 and thereby favors incorporation of COX4I2 into COX (Fukuda et al., 2007). Thus, reciprocal regulation of COX4I1 and COX4I2 protein levels represents an adaptive response that optimizes COX activity according to oxygen availability and protects tissues from oxidative damage.

### 4.2 COX6A

Similar to COX6B and NDUFA4 (see below), COX6A is thought to contribute to dimerization of COX monomers (Figure 3). Biochemical analyses of isolated bovine COX containing COX6A1 indicated that the dimeric state affects electron transfer and proton pumping (Lee & Kadenbach, 2001). In contrast, incorporation of the second isoform, COX6A2, into cardiac COX appears to have distinct functional consequences (Lee & Kadenbach, 2001). COX6A2 is a tissue specific isoform preferentially incorporated into COX of skeletal muscle (Figure 3), where it likely supports optimized OXPHOS in mature muscle fibers (Ye, Xie, et al., 2023). This leads presumably to an optimized oxidative phosphorylation in mature skeletal muscles (Ye, Xie, et al., 2023). Moreover, COX6A2 expression has been linked to the progression of diabetes, as increased COX6A2 levels in pancreatic cells can promote apoptosis, although the exact biochemical mechanisms remain unresolved (Kong et al., 2024; Shao et al., 2024). Overall, tissue specific expression of COX6A isoforms appears important for matching COX function to the energy demands of particular tissues.

### 4.3 COX6B

COX6B is one of the last nuclear encoded subunits to join cytochrome *c* oxidase during assembly (Fornuskova et al., 2010). Structural studies showed that COX6B promotes COX dimer formation and provides a platform for cytochrome *c* binding (Tsukihara et al., 1996). According to the assembly pathway, the absence of COX6B would be expected to result in accumulation of COX assembly intermediates. This has indeed been observed in fibroblasts and muscle cells from COX6B1 mutant patients, where COX intermediates accumulate, although some mature sized COX remains detectable (Abdulhag et al., 2015; Jennions et al., 2024; Massa et al., 2008). In contrast, complete knockout of COX6B1 in HEK293 cells causes a loss of fully assembled COX and, intriguingly, an absence of early assembly intermediates, suggesting that COX6B1 may also affect early biogenesis steps (Čunátová et al., 2024). Recent work by Čunátová et al. (Čunátová et al., 2026) expanded this view and proposed that COX6B1 (Figure 3) acts as a redox sensitive factor analogous to COA6, a copper delivery protein for COX2 (Maghool et al., 2019; Pacheu‐Grau et al., 2020; Soma et al., 2019). Both COA6 and COX6B1 contain a CX_9_C motif, hinting at potential redox regulated functions in copper handling during COX assembly(Čunátová et al., 2026; Maghool et al., 2019; Pacheu‐Grau et al., 2020; Soma et al., 2019). Although a direct role of COX6B1 in copper delivery remains to be demonstrated, it is likely that not all functions of COX6B1 have been uncovered.

The major COX6B isoform is COX6B1, which is ubiquitously expressed, whereas COX6B2 expression is largely restricted to testes (Figure 3) (Hüttemann, Jaradat, & Grossman, 2003). Altered COX6B2 levels in testes have been linked to reduced male fertility (Shimada et al., 2024). COX6B2 has also been identified as marker for testis cancer (Maxfield et al., 2015). Although the expression of COX6B2 has been described as testis specific (Hüttemann, Jaradat, & Grossman, 2003), it is also aberrantly expressed in several non‐testis cancers, where it has been associated with enhanced OXPHOS, increased tumor cell proliferation, and metastasis, for example in pancreatic ductal adenocarcinoma and lung adenocarcinoma (Nie et al., 2020). The same has been reported upon increased levels of COX6BI2 in lung cells during the progression of adenocarcinomas (Cheng et al., 2020). Mechanistically, an IGF2BP3–COX6B2 axis has been proposed (Lin et al., 2023): IGF2BP3 (insulin‐like growth factor 2 mRNA binding protein 3) as an mRNA binding protein, acting as a posttranscriptional regulator of gene expression within human cells and has been linked to tumor cell proliferation (Palanichamy et al., 2016; Zhou et al., 2017). The RNA binding protein IGF2BP3 seems to stabilize the COX6B2 mRNA, promoting COX6B2 expression and metabolic reprogramming of non‐small cell lung cancer (NSCLC) cells (Figure 3). Although, the binding of IGF2BP3 to the COX6B2 mRNA has been shown (Lin et al., 2023), IGF2BP1–3 are also known to localize partly to mitochondria (Cruz‐Zaragoza et al., 2021), but whether they also interact directly with COX6B2 protein in mitochondria remains unclear and warrants further investigation. Hence, the COX6B isoforms could present a metabolic link between COX activity and cellular gene expression.

### 4.4 COX7A

COX7A exists as three isoforms (Figure 3) with distinct tissue‐specific expression patterns. COX7A1 is predominantly expressed in muscle, whereas COX7A2 is present in most tissues with highest levels in kidney and liver (Pham et al., 2024). Both isoforms have been implicated in regulating mitochondrial oxidative capacity (Hüttemann, Klewer, et al., 2012; Lee et al., 2012), and loss of COX7A1 in mice leads to cardiomyopathy (Hüttemann, Klewer, et al., 2012) and altered muscle angiogenesis (Lee et al., 2012), underscoring its tissue‐specific role in muscle and heart.

The third isoform, COX7A2L, was originally implicated in the formation of respiratory chain supercomplexes and consequently named SCAF1 (supercomplex assembly factor 1) (Figure 3) (Lapuente‐Brun et al., 2013). SCAF1 exists as long and short isoforms in different tissues (Lapuente‐Brun et al., 2013). However, the role of SCAF1 in supercomplex formation and regulation remains controversial, as different studies have reported conflicting results regarding its necessity and function (Barrientos & Ugalde, 2013; Ikeda et al., 2013; Lapuente‐Brun et al., 2013; Mourier et al., 2014). Thus, the precise physiological role of SCAF1 (COX7A2L) in SC assembly and respiratory regulation is still unresolved.

### 4.5 COX7B

In humans, COX7B is expressed as two isoforms, COX7B1 and COX7B2 (Figure 3) (Blomme et al., 2006; Čunátová et al., 2026; Little et al., 2010). Very little is known about their differential expression, tissue distribution, and regulation. According to The Human Protein Atlas (https://www.proteinatlas.org), COX7B1 is broadly expressed in human tissues, whereas COX7B2 appears to be most abundant in testis. A polymorphism in the COX7B gene has been associated with an increased risk of nasopharyngeal carcinoma (Hui et al., 2004), suggesting that COX7B variants may modulate cancer susceptibility. However, more work is needed to determine whether differential COX7B1/COX7B2 expression reflects distinct COX activity requirements in specific tissues.

### 4.6 COX8

Two functional COX8 isoforms, COX8A and COX8C, are present in human tissues (Hüttemann, Schmidt, & Grossman, 2003; Rizzuto et al., 1989). A third isoform, COX8B, has become a pseudogene in humans (Figure 3) (Goldberg et al., 2003). Little is known about metabolic regulation of COX8 isoform expression. Current knowledge is limited to tissue distribution: COX8A is expressed in most tissues (Rizzuto et al., 1989), whereas COX8C expression appears restricted to testis, pancreas, and placenta (Kadenbach & Hüttemann, 2015).

### 4.7 NDUFA4

NDUFA4 is the most recently recognized structural subunit of cytochrome *c* oxidase (Balsa et al., 2012). It's relatively late re‐classification from a complex I to a COX subunit was most likely due to its loose association with the enzyme under stringent purification conditions. Zong and coworkers, provided structural information about the I + III_2_ + IV_1_ super‐complex at 3.3 A (Zong et al., 2018), NDUFA4 is positioned between the COX homodimer, and loss of NDUFA4 causes isolated COX deficiency (Pitceathly et al., 2013). A recent study by Nguyen et al. (2026) extended the COX super‐complex assembly model and according to structural analysis acts HIGD2A, former described as COX3 interactor (Hock et al., 2020; Timón‐Gómez, Garlich, et al., 2020), in super‐complex assembly. Additionally, HIGD2A has been described to be involved in the coordinated assembly of the COX1 and COX2 module into super‐complexes (Timón‐Gómez, Bartley‐Dier, et al., 2020; Timón‐Gómez, Garlich, et al., 2020). Interestingly, HIGD2A also effects the stability of NDUFA4 (Hock et al., 2020). Nguyen et al. (2026) recently implemented the model that HIGD2A serves as a placeholder for NDUFA4, while the COX1‐, COX2‐ and COX3‐module are assembled at a super‐complex intermediate (Figure 2).

However, the role of NDUFA4 appears to extend beyond its structural contribution to COX. NDUFA4 and its paralog NDUFA4L2 have been implicated in the modulation of PI3K/Akt signaling and are involved in tumor progression (Figure 3) (Clark et al., 2025; Ye, Wang, et al., 2023). NDUFA4 is broadly expressed, whereas NDUFA4L2 is preferentially induced in cancer and under hypoxic conditions, in part via HIF‐1 dependent transcriptional regulation (Fukuda et al., 2007; Lei et al., 2017; Pan et al., 2022; Reece et al., 2014), hence NDUFAL2 gets highly expressed in oxygen‐sensing glomus cells (Timón‐Gómez et al., 2022).

A third paralog of NDUFA4 (C15orf48) has been implemented to be involved in inflammation and spermatogenesis (Figure 3) (Clayton et al., 2021; Endou et al., 2020; Takakura et al., 2024). However, the precise mode of action, especially in regard to COX maturation and supercomplex assembly needs to be elucidated. To date, most insights into NDUFA4 dependent signaling come from cancer models, and it remains to be clarified whether NDUFA4 similarly modulates signaling pathways in healthy tissues.

## 5 CONCLUSIONS AND PERSPECTIVE

COX assembly proceeds through pre‐assembly modules in which core subunits (COX1, COX2, COX3) are matured. This assembly process can act as a control point that links mitochondrial electron transport to the energetic and redox demands of cellular metabolism. Especially, tissue‐ and condition‐specific isoforms of COX subunits provide a regulatory layer that fine‐tunes enzyme kinetics, oxygen affinity, and allosteric regulation by ATP/ADP to match variable energy needs and oxygen availability, aligning COX activity with the broader metabolic program of each cell. By modulating COX content, assembly efficiency, and isoform composition, cells can dynamically reconfigure respiratory chain capacity, redox poise, and ROS signaling, thereby supporting adaptation to developmental cues, workload changes, and environmental stress. To fully understand the involvement of COX at the level of whole‐cell metabolism, the investigation of different isoforms and tissue‐specific expression must be focused.

## AUTHOR CONTRIBUTIONS

**Sven Dennerlein:** Writing – original draft; writing – review and editing. **Peter Rehling:** Writing – review and editing.

## CONFLICT OF INTEREST STATEMENT

The authors declare no competing or financial interests.

## Data Availability

The data that support the findings of this study are available on request from the corresponding author. The data are not publicly available due to privacy or ethical restrictions.
